# 

**Published:** 1884-09

**Authors:** 


					﻿Vol. IV.
SEPTEMBER, 1884.
NO. 9.
THE
pDMEOPATHlC
A MONTHLY JOURNAL OF MEDICAL SCIENCE.
“ Seek the Truth: come whence it may, cost what it will."
IE. CR LEE, Js/L. I?.,
EDITOR.
CONTENTS:
(See inside of Cover.)
PHILADELPHIA:
No. 3109 CHESTNUT STREET.
JTEW YOUK:
A. L. CHATTERTON, PUBLISHING COMPANY, Publisher’sJAgents.
X.O3STUO3T:
ALFRED HEATH & CO., Sole Agents for Great Britain,
114 Ebury Street, Eaton Square.
Yearly Subscription, $2.50, invariably in advance.
Entered at the Philadelphia Post-Office as Second-Class Mail Matter.
				

